# A new non-invasive graphical method for quantification of cerebral blood flow with[$^{123}\hbox {I}$] IMP

**DOI:** 10.1007/s12149-018-1282-8

**Published:** 2018-07-25

**Authors:** Masashi Kameyama, Kiyotaka Watanabe

**Affiliations:** 1 Department of Diagnostic Radiology, Tokyo Metropolitan Geriatric Hospital and Institute of Gerontology, 35-2 Sakae-cho, Itabashi-ku, Tokyo, 173-0015 Japan; 2 Division of Nuclear Medicine, Department of Radiology, School of Medicine, Keio University, 35 Shinanomachi, Shinjuku-ku, Tokyo, 160-8582 Japan; 3 Department of Product Plannning, Nihon Medi-Physics Co. Ltd., 3-4-10 Shinsuna, Koto-ku, Tokyo, 136-0075 Japan

**Keywords:** Cerebral blood flow (CBF), Quantification, [$^{123}\hbox {I}$] *N*-isopropyl-*p*-iodoamphetamine (^123^I-IMP), Single photon emission computed tomography (SPECT), Kameyama’s method

## Abstract

**Objective:**

[$^{123}\hbox {I}$] *N*-isopropyl-*p*-iodoamphetamine ($^{123}\hbox {I}$-IMP) is an ideal perfusion tracer for single photon emission computed tomography, which shows good linearity between cerebral blood flow (CBF) and accumulation. However, quantification of CBF using $^{123}\hbox {I}$-IMP without arterial blood sampling has been challenging, with previous methods requiring empirically obtained regression formulae to estimate CBF. Furthermore, the CBF value obtained via some of the previous methods would be affected by the clearance rate of $^{123}\hbox {I}$-IMP from the lungs. This paper introduces a new non-invasive quantification method for CBF using $^{123}\hbox {I}$-IMP and dynamic planar images.

**Methods:**

We have developed a theory based on Microsphere model. This method does not involve regression formulae for estimation and allows for direct measurement of CBF, considering the clearance rate of $^{123}\hbox {I}$-IMP from the lungs. The study method is executed as easily as conventional Graph-Plot method. We compared the CBF values obtained by our study method and the established autoradiograph (ARG) method.

**Results:**

CBF values obtained using the new method demonstrated significant correlation with values determined using ARG method.

**Conclusions:**

The novel method described presents a reliable and more simple way of determining CBF when compared to current methods.

## Introduction

For quantification of cerebral blood flow (CBF) with single photon emission computed tomography (SPECT), [$^{123}\hbox {I}$] *N*-isopropyl-*p*-iodoamphetamine ($^{123}\hbox {I}$-IMP) is an ideal tracer as it has an advantage over other brain perfusion SPECT tracers, such as $^{99\rm {m}}$Tc-hexamethylpropyleneamine oxime ($^{99\rm {m}}$Tc-HMPAO) and $^{99\rm{m}}$Tc-ethyl cysteinate dimer ($^{99\rm {m}}$Tc-ECD), in that $^{123}\hbox {I}$-IMP does not suffer underestimation of regional cerebral blood flow (rCBF) at high flow values [1]. $^{123}\hbox {I}$-IMP shows retention in brain tissue by nonspecific, high-capacity, low-affinity amine binding sites within the brain after the initial uptake in the lung endothelium [2, 3].

The Microsphere model [4–6] is the simplest model for CBF estimation. However, the model assumes that the first-pass extraction (*E*) of tracer is high and efflux from the brain tissue is negligible. Fortunately, the *E* of $^{123}\hbox {I}$-IMP is extremely high [2] and its volume of distribution (=$K_1/k_2$) is around 30 [7]. As $^{123}\hbox {I}$-IMP concentration in brain tissue ($C_{\text {b}}(t)$) is limited during the early period, efflux from the brain tissue ($k_2 C_{\text {b}}(t)$) is negligible. Thus, $^{123}\hbox {I}$-IMP fulfills the assumptions of the Microsphere method if limited to the early phase of examination. However, the Microsphere method requires continuous or frequent arterial blood sampling to obtain input function. The Table Look Up method [7] and its simplification, the Autoradiograph (ARG) method [8] based on the one-tissue two-compartment model were developed to reduce the number of arterial blood samples to only one using standard input function, which was generated by combining the input functions from 12 subjects. Currently, the ARG method is the standard CBF quantification method using $^{123}\hbox {I}$-IMP.

The Gjedde–Patlak–Matsuda method, a non-invasive application of the Gjedde–Patlak plot [9–11] with $^{99\rm{m}}$Tc-HMPAO [12, 13] or $^{99\rm {m}}$Tc-ECD [14], which considers tracer activity in the aortic arch with planar acquisition as an input function, inspired investigation of a non-invasive CBF quantification method with $^{123}\hbox {I}$-IMP. The simple application of Gjedde–Patlak–Matsuda method to $^{123}\hbox {I}$-IMP was not successful owing to scatter radiation from the lungs into the region in aortic arch. The non-invasive microsphere (NIMS) method [15, 16] utilized pulmonary clearance of the tracer for the estimation of input function. However, this method was not pursued due to its requirement for complicated procedures such as measurement of right ventricular cardiac output.

**Fig. 1:**
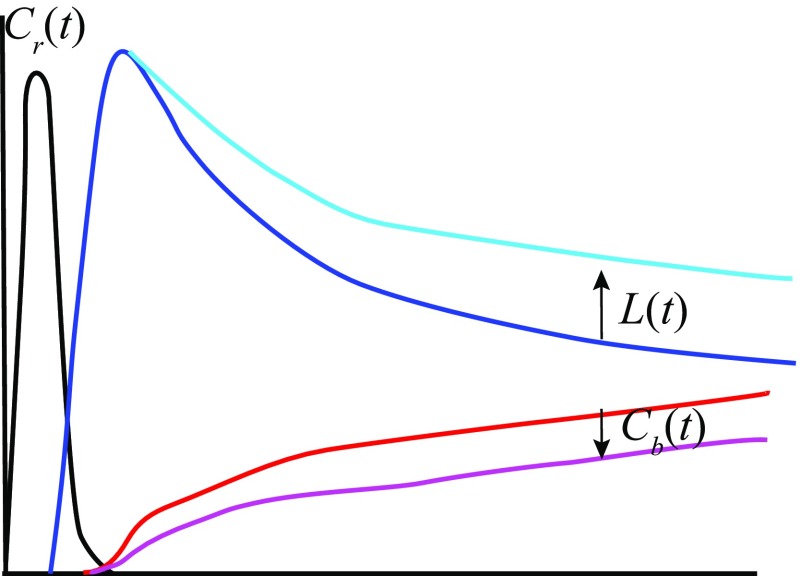
Schema of time–activity curve of pulmonary artery $C_{\text {r}}(t)$, lung *L*(*t*) and brain $C_{\text {b}}(t)$. Black line is $C_{\text {r}}(t)$, blue line is *L*(*t*) and red line is $C_{\text {b}}(t)$. light blue line is *L*(*t*) and pink line is $C_{\text {b}}(t)$ when $^{123}\hbox {I}$-IMP washout from lungs is slow. Note that even if the washout from lungs is slow, $C_{\text {r}}(t)$ does not change

The Graph-Plot (GP) method [17] is relatively simple to carry out. However, it contains several critical methodological flaws. The GP method is also an application of Gjedde–Patlak plot, which regards activity in the pulmonary artery trunk as an input function, instead of activity in the aortic arch as with the Gjedde–Patlak–Matsuda method. Input function for brain response function must be the activity in the artery of systemic circulation. Thus, use of pulmonary artery measurement as an input function is not appropriate. Though the cause has yet to be elucidated, the lung clearance of $^{123}\hbox {I}$-IMP showed great variation among patients [18]. Thus, the CBF value obtained via the GP method would be affected by the clearance rate of $^{123}\hbox {I}$-IMP from the lungs. For example, slow clearance from the lungs would reduce tracer activity in the artery ($C_{\text {a}}(t)$), as it would in the brain ($C_{\text {b}}(t)$), but would not affect tracer activity in the pulmonary artery ($C_{\text {r}}(t)$) (Fig. 1).

Furthermore, the GP method requires the application of a regression formula obtained via the ARG method to calculate an absolute value. Similarly, the Gjedde–Patlak–Matsuda method also involves a regression formula determined by the Kanno–Lassen method [19]. The simple non-invasive $^{123}\hbox {I}$-IMP quantification (SIMS) method that reduces the burden of NIMS also requires a regression formula to translate their SIMS index to input function from arterial blood sampling [20].

Many non-invasive methods for the calculation of CBF make use of regression formulae for estimation (Table 1). However, regression formulae obtained empirically generally amplify errors and reduce reliability of the system, because they contain dispersion and sometimes the formulae themselves are not appropriate. For example, the linear regression equation between HbA1c and average glucose by A1c-Derived Average Glucose (ADAG) study, $\text {AG} \mathrm{(mg/dL)}= 28.7 \times HbA1c (\%) - 46.7$ [21], is empirical and contains dispersion which is mainly due to between-patient variation in derived mean RBC age. We have just proposed a theoretical approach including mean RBC age, $\displaystyle HbA1c \simeq \frac{M_{\text {RBC}} k_{\text {g}}\mathrm{AG} }{1 + \frac{2}{3} M_{\text {RBC}} k_{\text {g}}\mathrm{AG} }$ [22]. Thus, equations obtained empirically using regression analysis are less reliable, and there is no evidence that the true relationship is linear.

Therefore, a new, non-invasive CBF measurement method using $^{123}\hbox {I}$-IMP but not requiring regression analysis has been long sought for, a problem solved by the appropriate application of mathematics in the method proposed in this article.

**Table 1:** Summary of quantification methods using $^{123}\hbox {I}$-IMP

Method	Invasion	Empirical regression formulae	Model	Remarks
Kameyama (2018)	None	None	Microsphere	This study
Microsphere (1982) [4]	Continuous ABS	None	Microsphere	
Yokoi plot (1993) [23]	Continuous ABS	None	1 tissue	Dynamic SPECT
Table lookup (1994) [7]	1 ABS	None	1 tissue	2 $\times$ SPECT, standard IF
ARG (1994) [8]	1 ABS	None	1 tissue	Fixed DV, standard IF
NIMS (1997) [15]	None	Cardiac output	Microsphere	Too complicated
GP (2002) [17]	None	CBF from ARG	Gjedde–Patlak	IF: pulmonary artery
SIMS (2016) [20]	None	Continuous ABS	Microsphere	Simplified NIMS
SIARG (2018) [24]	None	Continuous ABS	1 tissue	SIMS derivative

*ABS* arterial blood sampling, *IF* input function, *DV* distribution volume ($=K_1/k_2$)

## Materials and methods

### Mathematical theory

The Microsphere method assumes that the brain absorbs all tracer from the blood, so that first-pass extraction (*E*) is 1 and $k_2$, the rate constant which represents tracer movement from brain tissue to arterial plasma, can be ignored.

1 $$\begin{aligned} C_{\text {b}}(t) = F \int _0^t C_{\text {a}}(\tau )\lambda \,{\text {d}}\tau \end{aligned}$$

where $C_{\text {b}}(t)$ (Bq/g) is $^{123}\hbox {I}$-IMP radioactivity in the brain, $C_{\text {a}}(t)$ (Bq/mL) is concentration of $^{123}\hbox {I}$-IMP in arterial blood, *F* (mL/g/min) is CBF, and $\lambda$ is the lipophilic fraction of $^{123}\hbox {I}$-IMP, considering the metabolism of $^{123}\hbox {I}$-IMP. $\lambda$ is assumed to be 0.8 according to the measurement of octanol extraction [7].

Fick’s principle [25, 26] yields the following equation:

2 $$\begin{aligned} L(t) = P \left( \int _0^t C_{\text {r}}(\tau ) \,{\text {d}}\tau - \int _0^t C_{\text {a}}(\tau ) \,{\text {d}}\tau \right) \end{aligned}$$

where *L*(*t*) (Bq) is the total amount of $^{123}\hbox {I}$-IMP in the lungs, *P* (mL/min) is cardiac output from right ventricle, and $C_{\text {r}}(t)$ (Bq/mL) is concentration of $^{123}\hbox {I}$-IMP in the pulmonary artery.

Substituting Eq. (2) into Eq. (1) yields

3 $$\begin{aligned} C_{\text {b}}(t) = F\lambda \left( \int _0^t C_{\text {r}}(\tau ) \,{\text {d}}\tau - L(t)/P \right) \end{aligned}$$

Equation (3) divided by $\lambda L(t)$ yields

4 $$\begin{aligned} \frac{C_{\text {b}}(t)}{\lambda L(t)}= F \frac{\int _0^t C_{\text {r}}(\tau ) \,{\text {d}}\tau }{L(t)} - \frac{F}{P} \end{aligned}$$

When $C_{\text {b}}(t)/(\lambda L(t))$ is plotted on the *y*-axis and $\int _0^t C_{\text {r}}(\tau ) {\text {d}}\tau /L(t)$ is plotted on the *x*-axis, the slope of the line will represent *F*.

Equations (5) and (6) illustrate that imaging of a portion of the lungs is sufficient to calculate *F*.

5 $$\begin{aligned} l(t) = \alpha L(t) \end{aligned}$$

where *l*(*t*) (Bq) denotes the amount of the tracer in the visible portion $\alpha$ of the lungs.

6 $$\begin{aligned} \frac{C_{\text {b}}(t)}{\lambda l(t)} = F \frac{\int _0^t C_{\text {r}}(\tau ) \,{\text {d}}\tau }{l(t)} - \frac{F}{\alpha P} \end{aligned}$$

### Data acquisition

Fifty-nine patients at Sapporo Shuyukai Hospital, Japan, underwent cerebral blood flow SPECT for clinical reasons in September 2013 and from January 2015 to October 2015 and CBF quantification was undertaken using both ARG and GP methods. GP method was executed by a software package (AZE VirtualPlace Hayabusa^®^, AZE, Tokyo, Japan). ARG method was executed by software on a SPECT console.

The data were retrospectively analyzed. The committee in the hospital approved the use of de-identified data from these patients including dynamic planar images and the results of ARG and GP methods. The hospital provided the data under the contract between the hospital and Nihon Medi-Physics. For this type of retrospective study, written consents were waived. Dynamic front planar scans (2 s/frame, 60 frames, 4.4181 mm/pixel) were acquired by a $\gamma$ camera (GE Infinia 3, GE Healthcare, Waukesha, WI, USA) with an ELEGP collimator from the point of intra-venous bolus administration of 222 MBq $^{123}\hbox {I}$-IMP. Arterial blood sampling occurred 10 min after the $^{123}\hbox {I}$-IMP injection. SPECT images were acquired for 20 min commencing 20 min after the injection.

### Data analysis

**Fig. 2:**
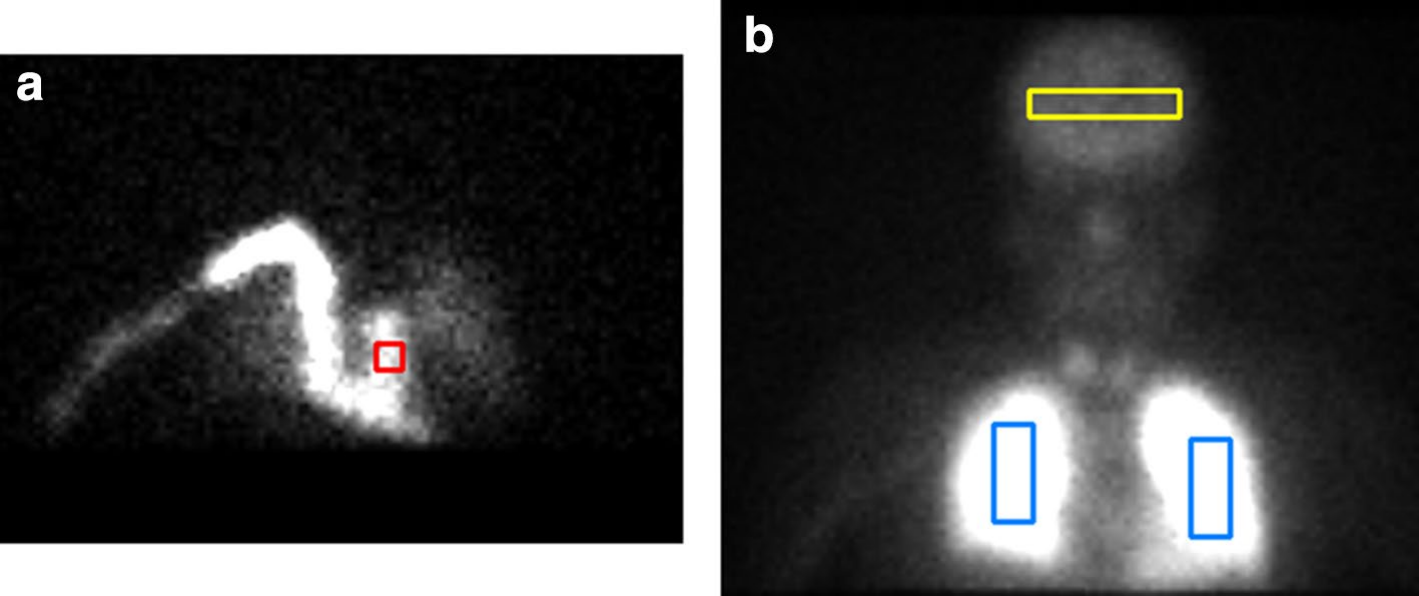
An example of the regions of interest; pulmonary artery trunk (**a** red), brain (**b** yellow) and lungs (**b** blue)

**Fig. 3:**
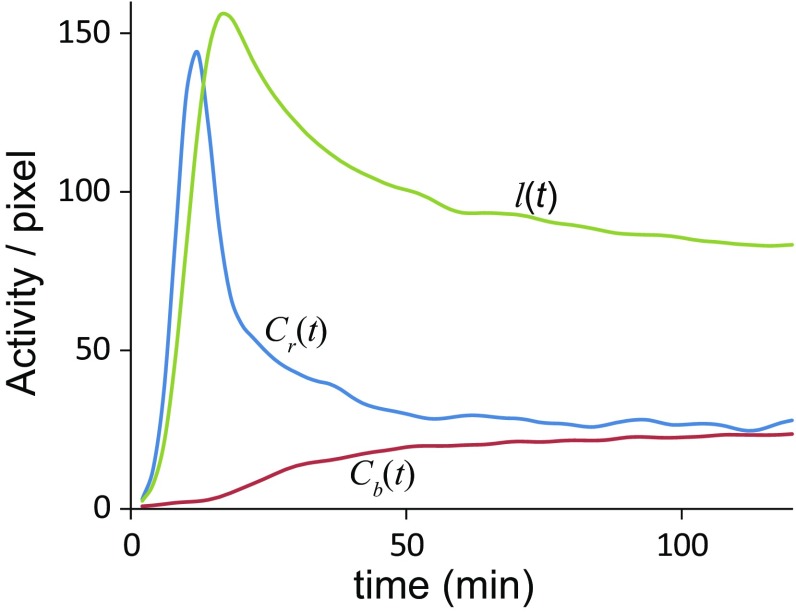
An example of time–activity curves of the three regions of interests; pulmonary artery trunk ($C_{\text {r}}(t)$), brain ($C_{\text {b}}(t)$) and lungs (*l*(*t*))

Data were analyzed on a standard spreadsheet software (Excel^®^ 2016, Microsoft Corporation, Redmond, WA, USA). Four regions of interest (ROIs) were selected on planar images. One on the pulmonary artery trunk, one on the brain and one on both lungs (Fig. 2). The GP method also utilizes ROIs in the brain and pulmonary artery trunk [17]. Time–activity curves (TACs) were derived from these four ROIs (Fig. 3).

We have obtained the mCBF value of ARG method from the same slice of CBF map as GP method and the study method.

**Fig. 4:**
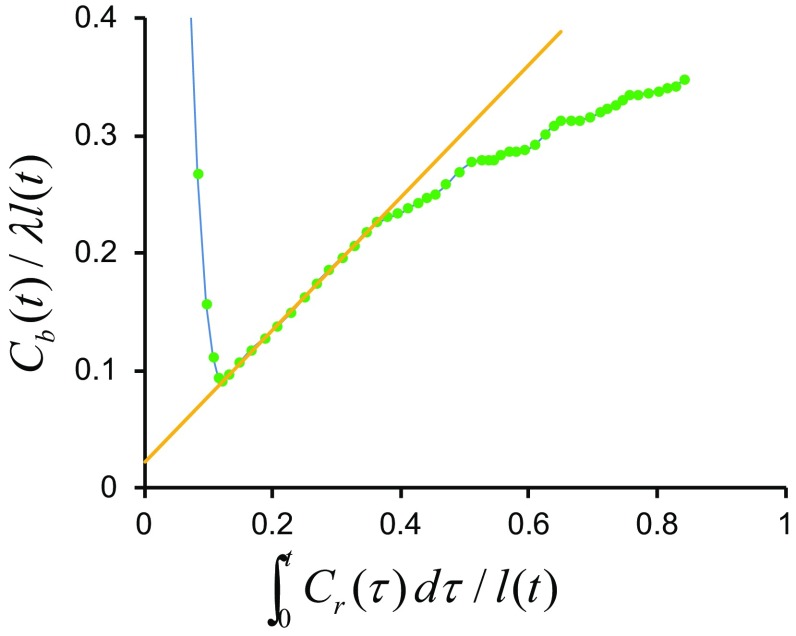
An example of plotting $\left( \int _0^t C_{\text {r}}(\tau ) \,{\text {d}}\tau / l(t) , C_{\text {b}}(t) / \lambda l(t) \right)$

The TACs calculated above were smoothed by low-pass filter to reduce statistical noise. Time zero-adjustment was then performed for time points where activity rose sharply [12, 17]. $\displaystyle \left( \frac{\int _0^t C_{\text {r}}(\tau ) \,{\text {d}}\tau }{l(t)} , \frac{C_{\text {b}}(t)}{\lambda l(t)} \right)$ was plotted and the slope of the linear section was determined (Fig. 4).

## Results

**Fig. 5:**
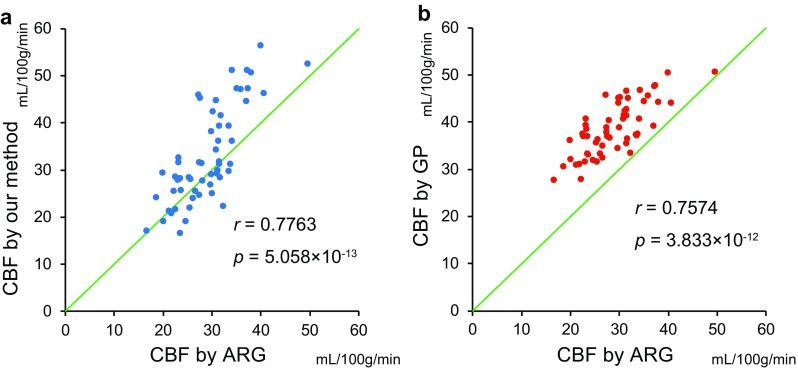
Relationship between ARG method and the study method/GP method. **a** Scatter plot of ARG and the study methods. **b** Scatter plot of ARG and GP methods. Green lines are identity lines

A significant correlation was observed between the CBF values obtained by the ARG method and the study method ($r=0.7763$, $p=5.058\times 10^{-13}$). Similarly, a significant correlation was found between figures derived via the ARG and GP methods ($r=0.7574$, $p=3.833\times 10^{-12}$) (Fig. 5).

However, CBF values determined by the GP method were generally higher than those calculated by the ARG method.

## Discussion

The aim of this study was to develop and determine the validity of a new non-invasive method for CBF quantification using $^{123}\hbox {I}$-IMP that would remove the need for arterial blood sampling and regression formulae for estimation. CBF values obtained using the novel method showed significant correlation with the ARG method, suggesting that this method would be of practical use.

We can estimate regional CBF values by distributing the acquired mCBF value according to mean brain counts and constructed perfusion images, like Gjedde–Patlak–Matsuda method [13] and the GP method [27].

### Study methods and NIMS

The novel method developed has many similarities with the NIMS method. However, modification of the original NIMS equation has allowed measurement of CBF without the need for measuring cardiac output from right ventricle (*P*) nor activity within entire lungs (*L*(*t*)).

Equations (7), (8) and (9) show the mathematical similarity between the study method and NIMS. Accumulation of the entire tracer dose in the lungs will occur within several seconds of injection ($T_1$ (min)).

7 $$\begin{aligned} P \int _0^{T_1} C_{\text {r}}(t) \,{\text {d}}t = D \; (> L_{\text {max}}), \end{aligned}$$

where *D* (Bq) is the injected dose and $L_{\text {max}}$ (Bq) is the peak *L*(*t*). Due to clearance from the lungs, $L_{\text {max}}$ should be smaller than *D*. Note that $C_{\text {r}}(t) =0 \; (t>T_1)$. Equation (7) is substituted into Eqs. (2) and (4)

8 $$\begin{aligned} L(t) = D - P\int _0^t C_{\text {a}}(\tau ) \,{\text {d}}\tau \; (t>T_1)\end{aligned}$$

The NIMS equation is as follows:

9 $$\begin{aligned} \int _0^t C_{\text {a}}(\tau ) \,{\text {d}}\tau = D \left( 1-\frac{L(t)}{L_{\text {max}}}\right) /P \end{aligned}$$

The study Eq. (8) and the NIMS Eq. (9) differ only in that *D* is substituted for $L_{\text {max}}$. It has been reported that $\left( 1-\frac{L(3)}{L_{\text {max}}}\right)$ [right side of Eq. (9)] consistently underestimates arterial blood sampling data $P\int _0^3 C_{\text {a}}(\tau ) \,{\text {d}}\tau /D$ [left side of the Eq. (9)] [16]. This is likely due to the use of $L_{\text {max}}$ instead of *D*. SIMS method [20] and SIARG method [24] suffer the same issue.

### Study method and the GP method

While the study equation and the GP equation appear similar, the underlying principles differ greatly.

10 $$\begin{aligned} \frac{C_{\text {b}}(t)}{C_{\text {r}}(t)}= F_{\text {GP}}\frac{ \int _0^t C_{\text {r}}(\tau ) {\text {d}}\tau }{C_{\text {r}}(t)} - K \; \left( K = \frac{F_{\text {GP}}L(t)}{P C_{\text {r}}(t)}\right) \end{aligned}$$

where $F_{\text {GP}}$ is the index value used in the GP equation to calculate CBF with the aid of a regression formula. The GP method is, in fact, an application of the Gjedde–Patlak plot. This method is flawed in that it does not consider tracer pharmacodynamics within the lungs and *K* is not a constant. Substituting $C_{\text {r}}(t)$ for $C_{\text {a}}(t)$ is not appropriate. Certainly we used $C_{\text {r}}(t)$, but it is not an input function in the study method.

Furthermore, the use of a regression formula in itself can be a source of error. CBF as determined by the GP method was higher than the values obtained via ARG, and this was especially so in instances of low flow, a result of regression analysis (Fig. 5). Also, the regression formula for each $\gamma$ camera needs to be determined separately as one regression formula cannot be applied to all. Despite these flaws, figures calculated using the GP equation were significantly correlated with values calculated using the more reliable ARG method. It is possible that, despite previous reports [18], the variability in clearance of $^{123}\hbox {I}$-IMP from the lungs was not great enough to influence the outcome of the equation. It should be noted that the ARG method also suffers over-simplification as it uses standard input function, which neglects variability in input functions.

The equations below aim to elucidate the relationship between $F_{\text {GP}}$ and *F*. The overall tracer uptake rate constant for the system has been shown to be [11]:

11 $$\begin{aligned} F_{\text {GP}}= & {} \frac{C_{\text {b}}(\infty )}{ \int _0^\infty C_{\text {r}}(t) \,{\text {d}}t} \end{aligned}$$

12 $$\begin{aligned} F= & {} \frac{C_{\text {b}}(\infty )}{ \int _0^\infty C_{\text {a}}(t) \,{\text {d}}t} \end{aligned}$$

Here, Eqs. (7) and (11) yield

13 $$\begin{aligned} F_{\text {GP}} = C_{\text {b}}(\infty ) P / D \end{aligned}$$

Equations (7) and (2) and $t=\infty$ yield

14 $$\begin{aligned} \int _0^\infty C_{\text {a}}(\tau ) \,{\text {d}}\tau = \frac{D - L(\infty )}{P}
\end{aligned}$$

Equation (14) makes Eq. (12) as follows:

15 $$\begin{aligned} F= \frac{C_{\text {b}}(\infty ) P}
{ D - L(\infty )} \end{aligned}$$

These Eqs. (13) and (15) yield

16 $$\begin{aligned} F = \frac{F_{\text {GP}} }{1-\kappa } 
\; (\kappa = L(\infty ) / D) \end{aligned}$$

where $\kappa$ is the fraction of tracer in the lungs. This shows mathematically that the GP equation overestimates input function due to its lack of consideration of tracer retained in the lungs, leading to underestimation of $F_{\text {GP}}$. However, as mentioned earlier, if the fraction of tracer retained in the lungs remains largely constant, the GP method will provide valid results.

### Limitations

The study method is based on the Microsphere model which assumes first-pass extraction (*E*) is 1 and neglects to consider back-diffusion. Certainly, extraction of $^{123}\hbox {I}$-IMP approaches 1 and $k_2$ of $^{123}\hbox {I}$-IMP is small; however, *E* is not 1 and $k_2$ is not 0, thus underestimation of *F* is possible.

The authors are aware that measurements taken from the pulmonary artery trunk include scatter radiation from the lungs. While subtraction of the scatter radiation would yield more accurate measurements, a reliable technique to correct TAC in this way could not be devised. Therefore, overestimation of tracer activity in the pulmonary artery trunk due to scatter radiation artifact is likely to cause underestimation of *F*.

We treated lipophilic fraction, $\lambda$ as a constant to make calculation simple, which is in fact a variable of time, reflecting the process of the metabolism of ^123^I-IMP. However, after quick conversion, lipophilic fraction seems relatively stable [7], and NIMS method also adopted a constant lipophilic fraction [16].

We assumed that lung tissue is everywhere the same (Eq. 5). We believe that the assumption would be fulfilled if the patient does not have an evident heterogeneous lung disease. Moreover, we have in fact confirmed the robustness of the study method by various types of lung ROIs (data not shown). However, we admit that patients with lung disease (e.g., a patient with her right lung damaged by tuberculosis) would not fulfill the assumption, although other methods that use standard input function, including ARG method cannot be applied to the patients with lung disease, either.

Finally, further study into the attenuation of planar images in the head versus the pulmonary artery trunk is required to correct for differences in attenuation.
